# Measurable benefits on brain activity from the practice of educational leisure

**DOI:** 10.3389/fnagi.2014.00040

**Published:** 2014-03-11

**Authors:** Carmen Requena, Verónica López

**Affiliations:** Chair “Aging at all Ages”, Departamento de Psicología, Universidad de LeónLeón, Spain

**Keywords:** leisure activities, old people, EEG, beta and alpha bands, compensation, retired people

## Abstract

Even if behavioral studies relate leisure practices to the preservation of memory in old persons, there is unsubstantial evidence of the import of leisure on brain activity.

**Aim:** This study was to compare the brain activity of elderly retired people who engage in different types of leisure activities.

**Methods**: Quasi-experimental study over a sample of 60 elderly, retired subjects distributed into three groups according to the leisure activities they practised: educational leisure (G1), memory games (G2), and card games (G3). Applied measures include the conceptual distinction between free time and leisure, the test of the organization of free time measuring 24 clock divisions, and EEG register during 12 word list memorizing.

**Results**: The results show that the type of leisure activity is associated with significant quantitative differences regarding the use of free time. G1 devotes more time to leisure activities than G2 (*p* = 0.007) and G3 (*p* = 0.034). G1 rests more actively than the other two groups (*p* = 0.001). The electrical localization of brain activity indicated a reverse tendency of activation according to the bands and groups.

**Discussion**: Engaging in educational leisure activities is a useful practice to protect healthy brain compensation strategies. Future longitudinal research may verify the causal relation between practicing educational leisure activities and functional brain aging.

## INTRODUCTION

The responsibilities of retired elderly people diminish at the same rate as the risk of cognitive deterioration increases if their daily routines are not occupied with new mental, social, and physical activities (Calero and Navarro, 2011). In studies measuring cognitive and cerebral activity, it appears that engaging in leisure activities is not only useful to prevent cognitive decline (Noack et al., 2009) but also, it prolongs adult autonomy and therefore, reduces public health costs (Infurna et al., 2011).

The “Bronx Aging Study” (Hall et al., 2009) associates prevention in impairment with the frequency of leisure activities, in particular activities such as reading, board games, playing musical instruments, and dancing are associated with enhanced mental performance. This is even the case after adjusting hazard variables such as age, gender, educational level, and basal cognitive state. Similar results were also obtained in another study (Verghese et al., 2003), in which around 50 individuals over 75 years old of age participated. This study showed that people who took part in leisure activities twice a week were significantly less likely to develop dementia. Likewise, another study on elderly people concluded that frequent participation in stimulating cognitive activities leads to a mean decrease in cognitive decline, which was 47% lower among individuals with greater cognitive activity (Willis et al., 2006).

A post-mortem analysis of participants from a long term longitudinal study provided evidence that mental stimulation in old age protects against memory loss (Wilson et al., 2007). It was found that a cognitive active person had 2.6 times less probability of developing dementia in old age than a cognitive inactive person at this age. This association persisted after normalizing for variables such as educational level, socioeconomic status, and participation in leisure activities initiated before and after retirement. In this sense, another study confirmed that an enriched environment during old age, with more opportunities for exercise, exploration or interacting with others, drastically reduces the loss of cognitive functionality (Lazarov et al., 2005).

Interestingly, in a study in which more than 6000 people were interviewed every 3 years over a total of 14 years, it was found that while individuals with a higher education performed better in the memory and thinking skills tests at the beginning of the study, the difference with less educated subjects became smaller over the course of the study (Wilson et al., 2009). This result suggests that the benefit of a higher education does not by itself reduce the risk of dementia but rather, other aspects protect from deterioration during old age, such as healthy lifestyle, economic status, and leisure activities. These data were confirmed by the study from Valenzuela et al. (2013) who found that the impairment rate was reduced by 50% in adults taking part in cognitive leisure activities.

It is significant that these functional results seem to be contradicted by studies of brain function using neuroimaging techniques such as magnetic resonance (Jones et al., 2011). The aging brain presents irreversible biological changes of cerebral activity that are systematically associated to functional decline. The problem is to explain how retired people can perform well in cognitive tasks when they are simultaneously being affected by the aging of their brains.

Two types of models have been used to explain the capacity of aged brains to compensate for the disruption to standard processing networks, implying the use of brain structures or networks that are not usually utilized by individuals whose brain remains intact (Cabeza et al., 2002a). In the first, the simplest form of cerebral compensation occurs when a more intensive use of the alternative network is associated with higher efficiency. This form of compensation is consistent with the model in which older people that perform best recruit additional areas of the brain, generally at the contralateral hemisphere from the one used by healthy brains (as proposed by Cabeza et al., 2002b).

Alternatively, different studies reported compensation reassignment that is not restricted to the contralateral hemisphere (Logan et al., 2002). In this regard, a study comparing the performance of young and old adults in a verbal memory test showed that the older group presented a more marked pattern of pre-frontal bilateral activity than the younger ones (Zarahn et al., 2007). Other studies along similar lines suggested that pre-frontal cortex activity tends to be less lateral in older people than in younger adults (Habeck et al., 2003). Although the alternative network could successfully support the performance of tasks, it is not as optimal as the primary network given that the results of the older adults were worse than those of the younger ones (Aurtenetxe et al., 2012). A simple analogy would be the use of a walking stick, which allows an older person to walk but not as well as another older person who does not need a walking aid.

Electroencephalogram (EEG) recordings represent another index of brain aging (Basar and Guntekin, 2012), particularly in terms of the insidious changes in fast bands. As age advances, alpha wave oscillations decrease during access and recovery of semantic information in frontal areas (Jensen et al., 2002). However, this evidence not only refers to long term memory but also to tasks involving working memory, since a decrease in the frequency peaks of the alpha band is mainly observed in the temporal, central bilateral, and posterior regions (Klimesch et al., 2008). Other studies have provided evidence that alpha rhythms decrease as verbal memory tasks become more difficult, particularly in occipital and parietal areas (Teplan et al., 2006). In a similar way, spectral EEG analyses manifest the association between the delay in the beta band and an increase in age. Studies into retention and complex cognitive tasks showed a smaller activation of frequency peaks in the beta band within the left frontal area in young adults (Jacobs and Kahana, 2010).

The aim of this research was to compare brain activity of alpha and beta bands in elderly retired people practicing leisure in their free time. Type, number, frequency, and duration of leisure activities are measured by the test of the organization of free time (TOFT) set up by the authors.

## METHODS

### SUBJECTS

The initial sample consisted of 72 subjects of whom eight were excluded because of non-compliance with study criteria and four due to the abnormal basal EEG recordings. The inclusion criteria were: individuals older than 65 years of age (see **Table 1**) with elementary school education and who have been practicing the same leisure activity for at least 3 years, as could be confirmed in records. The exclusion criteria applied were: elderly people with a medical history of neurological or psychiatric disease, or MMSE < 25/30.

**Table 1:** Distribution by age and gender of three groups.

	G1		G2		G3	
*N*	19		20		21	
	70,11 (±3,59)		71,61 (±3,32)		73,25 (±2.10)	
Ages/gender	M	F	M	F	M	F
	67	65	67	66	71	71
	68	66	68	67	71	72
	70	66	69	69	72	72
	70	67	72	70	72	72
	72	68	72	72	73	72
	76	70	72	72	73	72
	78	70	73	72	75	73
		70	73	73	75	73
		70	74	75	77	73
		70	75	80		74
		73				76
		76				79

### PROCEDURE

Subject recruitment was performed among elderly retired people enrolled in leisure activities offered by Day Centers in the city of León (Spain). All the study volunteers were called to a meeting where the conditions of participation were explained. Those interested completed a form providing their personal details and a contact telephone to arrange the assessment appointments. The test was applied over 40 min and all the subjects gave written informed consent before undertaking screening tests.

The subjects were distributed according to leisure activities defining leisure activity as the set of voluntary, unpaid activities with an educational goal or for entertainment, which require some kind of effort. Therefore, not all free time is leisure time (Foubert-Samier et al., 2012).

Group 1 (G1): life-long learning seminars. In this leisure activity different professionals teach and run chats or “tertulias” related to the disciplines of history, literature, sociology, and psychology with the objective of offering learning and personal growth opportunities through reflection and dialog with peers and teachers. Group 2 (G2): memory games. This leisure activity is lead by a psychologist and it consists of activities aimed at reinforcing the three main phases of the memory process: registration (e.g., visual acuity and stimulus discrimination), retention (retaining numbers and/or names), and recall (e.g., remembering lists by categories). Group 3 (G3): card games. This leisure activity involves organizing competitive card games between groups of four people to be played in pairs. The activity is supervised by a social educator.

The frequency of participating in the described leisure activities is twice weekly in 2 h sessions from October to May 2012.

#### Word memory task and EEG

Electroencephalogram recordings were obtained from subjects taking part in the study over 10 min, 4 min recording of the basal level and 6 min recording while the subject was in the experimental condition of memorizing a 12 word list (Wechsler-III Memory scale subtest; Wechsler, 2004). The memory task was performed consecutively four times through aural stimulation asking the subjects to try to remember as many words as possible in any order.

#### Measurements

**FIGURE 1:**
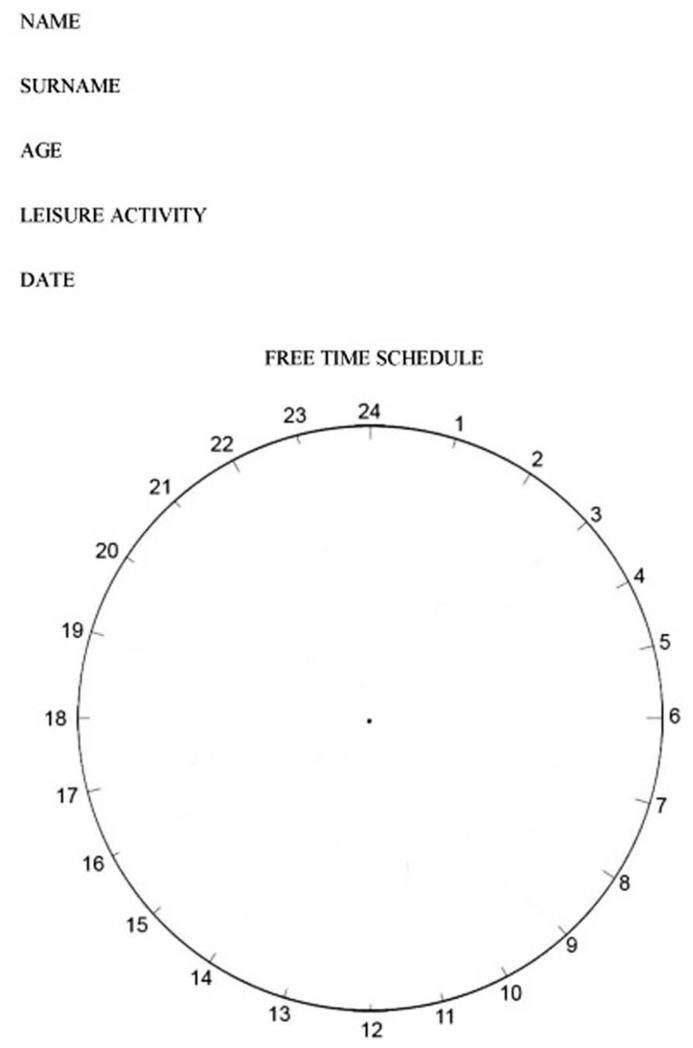
**Test of the organization of free time (TOFT)**.

***Test of the organization of free time.*** This test was created by the authors specifically for this research study. It consists of a circle that is divided into 24 spaces, representing the hours in a day. The variables that are quantified in this test are: (1) Clock divisions based on the free time occupied throughout the day; (2) Hours devoted to daily living activities (DLA); (3) hours dedicated to leisure activities; (4) Hours dedicated to rest. The subjects are instructed to represent on the clock the day of the week on which they have more free time, excluding Saturdays, and Sundays (please see **Figure 1**)

***Word memory list.*** This is a subscale of Wechsler-III memory (Wechsler, 2004). Once age and education level indexes were applied, for ages between 66 and 73 years, the number of words to be remembered were 3–4, while for an age equal or higher than 74, corresponded 2–3 to be remembered.

### ELECTROENCEPHALOGRAM

Electroencephalograms were recorded with a 32 channel NeuronicMedicid® apparatus using a standard 10–20 electrocap, keeping the impedance of all electrodes below 5 kΩ. An electrooculogram (EOG) was recorded with two pairs of leads in order to register horizontal and vertical eye movement. Data were recorded using a mastoid electrode as the reference and at a sampling rate of 1000 Hz. Amplifier frequency bands were set between 0.05–100.0 Hz.

#### Source localization

Low-resolution electromagnetic tomography (LORETA; Pascual-Marqui et al., 1994) was applied to the individual event-related potential (ERP) recording to identify the underlying electrical brain sources of scalp potentials. LORETA is a reverse solution method that calculates the three-dimensional distribution of neural generators in the brain as a current density value (A/m2) for a total of 2.394 voxels, with the constraint that neighboring voxels show maximal similarity.

Anatomical restrictions in brain volume were applied for cerebral electrical tomography (CET) calculations and an average brain template was used. The CET data were analyzed over time and tomography was calculated for each instance separately. Two source analyses models were defined by constraining the source to one anatomic compartment that was selected with the probabilistic brain atlas (PBA: Collins et al., 1994; Mazziotta et al., 2001) and Brodmann’s atlas (Mazziotta et al., 2001).

Electroencephalogram recordings were obtained in a soundproof room with dim lighting. The subjects were seated comfortably and they were instructed to stay awake, keep their eyes open, and avoid abrupt movements.

## RESULTS

### ANALYSIS OF VARIANCE

An ANOVA variance analysis was performed in order to evaluate the differences across groups due to leisure activities. *Post hoc* comparisons were performed using the Bonferroni method of multiple comparisons.

Descriptive data regarding the characteristics for the three groups studied are shown in **Table 2**, including how leisure time was organized, general memory, and word memory (WM).

**Table 2:** Descriptive data for measures TOFT, TMT, and WM.

	G1	G2	G3
***N***	19	20	21
**Gender**
		
M	7	9	9
F	12	11	12
**Age**	70,11 (±3,59)	71,61 (±3,32)	73,25 (±2.10)
**TOFT**
		
No. of divisions of the clock	11,2 (±1.61)	10,05 (±2.19)	8,24 (±1.32)
No. of activities	3,27 (±1.38)	2,32 (±0.94)	2,32 (±1.03)
DLA	5,87 (±2.32)	6,84 (±2.41)	5,88 (±1.98)
Leisure time	6,47 (±2.09)	4,37 (±2.36)	4,56 (±1.53)
Rest time	2,27 (±1.10)	3,58 (±1.42)	3,52 (±1.61)
			
**WM**	5.00 (±1.09)	4.14 (±1.45)	3.14 (±1.17)

*TOFT, test of the organization of free time; TMT, Trail Making Test; WM, word memory; DLA, daily living activities.*

#### Inter-group analyses

There were significant differences between the three groups of leisure activities and the TOTF values for the variable *clock divisions* (*F*_2,57_ = 10.80, *p* < 0.001), with *post hoc* differences between G1 and G2 (*p* = 0.001) and between G2 and G3 (*p* = 0.047). In the variable *hours dedicated to ADL* (*F*_2,57_ = 3.80, *p* < 0.028), *post hoc* differences were found between G1 and G3 (*p* = 0.032). For the variable *resting hours* (*F*_2,57_ = 16.52, *p* < 0.001), *post hoc* differences between the three groups could be observed (*p* = 0.001), while for the variable *leisure hours* (*F*_2,57_ = 5.72, *p* < 0.005), *post hoc* differences between G1 and G2 (*p* = 0.007) and G1 and G3 (*p* = 0.034) were evident.

There were significant differences between the three groups of leisure activities and the WM (*F*_2,57_ = 11.29, *p* < 0.001), with *post hoc* differences between G1 vs. G3 (*p* = 0.001), y G2 vs. G3, (*p* = 0.014).

**FIGURE 2:**
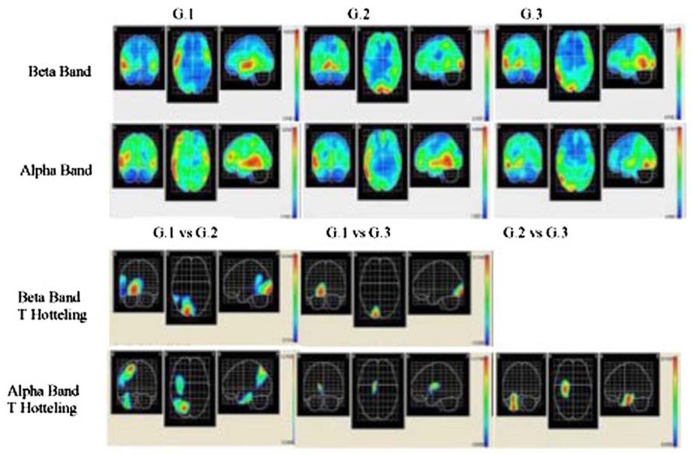
**Descriptive and comparative data of beta and alpha bands.** Two upper rows show cortical intensity projection (LORETA) mean maps obtained each group. Maximal intensity projection areas are displayed in red. Lower row show statistical mapping (SM) independent Hotelling T^2^ significant differences maps between groups.

Source analysis for each group was carried out, and the mean beta and alpha bands were calculated (LORETA) for G1, G2, and G3 during 12 word list memorizing The activated areas in the G1 beta band were the right superior temporal area, right medial temporal area, right insular, right pre-central region, and right lower frontal area, with the maximal activation area in the left lateral occipitotemporal area. In G2, the areas activated were the right and left lingual areas, and the right and left occipital poles, while the right lingual area was that most strongly activated. The activated areas in G3 were the right temporal medial region, right lingual gyrus, and the right inferior temporal area, while the right medial temporal area was the region most strongly activated (see **Figure 2**).

The results of measuring the alpha band in the G1 group show that the areas activated were the right medial temporal area, right lower temporal area, right and left high temporal region, left pre-central area, and the right medial frontal area, with the strongest activation in the right medial temporal area. The activated areas in G3 were the right lower temporal area and right lateral occipitotemporal region, although the most strongly activated was the right medial temporal area (see **Figure 2**).

In each group, voxel-by-voxel statistical mapping (SM) was computed to find the mean differences in the activated sources between groups. An independent Hotteling’s T^2^ test for multiple inter-group comparison (G1 vs. G2, G1 vs. G3, and G2 vs. G3) was applied within each band, considering degrees of freedom, and threshold values of *p* < 0.05; *p* < 0.01; and *p* < 0.001 (see **Table 3**; **Figure 2**)

**Table 3:** Summary of significant differences between groups with respect to maximal intensity projection areas during word memorizing list.

	AAL	BA	X	Y	Z	T^2^Hotelling	Mean		Mean		Mean	
							G1	G2	G1	G3	G2	G3
**Beta band**	Right lingual area	18	72	64	180
	17.9687^*^	2.2709	6.3441				
	Right occipital pole	17	72	68	196	18.3139^*^	0.4392	1.3834				
	Right inferior temporal area		28	96	152	13.7555^*^			0.6473	1.2389		
**Alpha band**	Right superior parietal lobe	7	68	132	164	13.7570^**^	0.2585	0.4152				
	Right caudate nucleus		80	84	88	11.1170^*^			0.0234	0.0263		
	Right parahippocampal gyrus	20	56	52	100	26.1634^*^					0.2881	0.2133

* *p* < 0.05, **p < 0.001.

For Beta band. word memorizing condition: G1 vs. G2 T^2^ (3–37) for α = 0.01 (18.3140); G1 vs. G3 T^2^ (3–38) for α = 0.05 (13.7560). For Alpha band. word memorizing**condition: G1 vs. G2 T^2^ (3–37) for α = 0.05 (13.7570); G1 vs. G3 T^2^ (3–38) for α = 0.05 (11.1170); G2 vs. G3 T^2^ (3–41) for α = 0.001 (26.1634). AAL = Anatomical label corresponding to PBA; BA = Brodmann areas; x, y, z = co-ordinates from PBA in three spatial axes; L = Left, R = Right; *p < 0.05; and **p < 0.001.

## DISCUSSION

The results from this study indicate that the type of leisure activity is associated with significant quantitative differences regarding the use of free time and localization of electrical brain activity, particularly between subjects belonging to the G1 group and subjects from G2 or G3.

Differences between the study groups were evident in the variable *time management* in the TOFT. While G1 subjects describe free time occupation with detail (type of activity, frequency, and timetable specification), subjects from G2 and G3 distribute the hours of the clock broadly. This TOFT difference reflects the variability and dynamic nature of the everyday life of subjects in G1 in contrast to the routine life of the subjects in G2 and G3. This result is confirmed by the fact that G1 subjects dedicate less time to resting and they conceive this in an active way. This means that they dedicate that time to intellectual activities (e.g., reading the newspaper, completing a Sudoku, word searches) or physical (e.g., walking) and social (visit family and friends) activities. In the other two groups (G2 and G3), *resting time *occupies a larger number of hours than leisure and DLA time, and leisure is conceived in a passive way (e.g., watching TV or having a nap). In this sense, some studies aimed at evaluating the healthcare benefits demonstrated that while an active lifestyle helps protect against deterioration, tendency to a sedentary lifestyle increases disability, and other co-morbidities (Fried et al., 2004; Iwasa et al., 2012). Moreover, another study showed that the preference for these types of activities entails less social life and more severe solitude (Schmitt et al., 2010). Therefore, subjects from the G1 group also adopt a more active lifestyle in all their free time, including rest.

Avoiding memory loss has also been associated not only with the variety of activities undertaken but also, with the frequency and type of activities (Infurna et al., 2011). In this sense, the first comprehensive review of research into memory preservation highlighted the role of complex and mentally stimulating educational activities as a form of compensating for the memory deterioration in aging. After analysing 29,000 people in 22 studies, it was concluded that engaging in educational and complex activities reduces the risk of dementia by almost half (Valenzuela et al., 2013). Subjects from the G1 group took part in activities that require learning new complex and difficult skills, as opposed to the habitual activities chosen by subjects in G2 (training for memory preservation) and G3 (card games).

The EEG results indicated a reverse tendency of activation according to the bands and groups. In G1 subjects, beta band activation was located in the right hemisphere, whereas in the other two groups activation was bilateral. These results did not correspond to those from healthy individuals, as for verbal memory tasks the band activated is localized to the left frontal area (Habeck et al., 2003; Zarahn et al., 2007). The localization of the alpha band corresponded to an expected pattern in the G1 group, since activation of this band in the bilateral temporal area in verbal memory tasks has been identified (Jacobs and Kahana, 2010), corresponding with the experimental condition designed for EEG recording in our study. By contrast, in G2 and G3 subjects activation was localized in temporal areas and it is concentrated in the right hemisphere.

Topographic localization of beta and alpha bands is associated to different brain regions depending on the groups. In G1 subjects these bands were localized to anterior areas, in G2 to middle areas and in G3 to posterior areas. Nevertheless, the descriptive analysis showed a coincidence in that the right temporal medial region is where maximum activation both in beta and in alpha bands is reached in all three study groups. The significant differences between groups support the finding that the highest activation of these bands is in the right hemisphere. The sites of maximum significance according to the number of voxels (see T^2^ Hotelling in **Table 3**) are located in medial and posterior areas in this hemisphere.

Therefore, since processing of verbal tasks does not correspond with the main activation of the right hemisphere, the data from our study reveals a form of compensation that is consistent with the model where older people recruit additional areas from the contra-lateral hemisphere to optimize performance in cognitive tasks [as proposed by Cabeza et al. (2002a)]. This model is consistent with the data regarding the number of words that both G2 and G3 subjects remember (three words), as well as those by G1 subjects (four words). Furthermore, if we consider the comparative results between groups, we observe that the highest brain activation (See Hotelling T^2^ in **Figure 1**) corresponds to G2 and G3 subjects as opposed to G1. We conclude that G1 subjects have greater cerebral efficiency in the test, that is, they required less activation to obtain a more efficacy (Jones et al., 2011). Therefore, taking part in educational leisure activities (G1) seems to intervene in the brain aging process.

There are certain limitations associated with our study, the design of which is cross-sectional, such that we can’t establish the sequence of events associated with the study variables. For future research it would be convenient to use a longitudinal sequential design that would allow us to verify the causal relation between practicing leisure activities and functional brain aging. If these results are confirmed, they will support the recommendation that taking part in leisure activities helps reduce risk in aging brains.

## Conflict of Interest Statement

The author declares that the research was conducted in the absence of any commercial or financial relationships that could be construed as a potential conflict of interest.
